# A Flexible Electret Membrane with Persistent Electrostatic Effect and Resistance to Harsh Environment for Energy Harvesting

**DOI:** 10.1038/s41598-017-07747-y

**Published:** 2017-08-16

**Authors:** Xiao Huiming, Chen Gangjin, Chen Xumin, Chen Zhi

**Affiliations:** 0000 0000 9804 6672 grid.411963.8 Lab. of Electret and Its Application, Hangzhou Dianzi University, Hangzhou, 310018 China

## Abstract

A novel flexible electret membrane, exhibiting persistent electrostatic effect, distinctive temperature stability and outstanding capability of resistance to harsh environment and fatigue, is demonstrated by experiment. Its excellent electret performance is correlated to the synergy of three factors, which are space charge injection, dipole orientation and interfacial polarization according to the analysis of charge storage mechanism. This electret membrane is provided with sandwich configuration PTFE/THV/PTFE, prepared by hot pressing method and thermal charging technology. After wiped its surface with alcohol, its surface potential declines to zero from −550 V, then recovers rapidly to −310 V and finally maintains constant for 800 hours, which shows that its electret performance distinctly precedes traditional electret material such as single PTFE, FEP electret membrane. The measurement of thermal stimulating potentials displays that its surface potential reaches maximum about 5 times initial value at 125 °C. A micro-vibration energy harvester is assembled with this membrane. Its maximum output power reaches 4.66 μW at tapping frequency 5 Hz and keeps stable during over 2000 tapping tests within 100 days, which indicates the long-life service and resistance to harsh environment and fatigue of this electret membrane.

## Introduction

An electret is a functional dielectric material exhibiting a quasi-permanent external electrostatic field. Electrets are booming as much interest as they do for both theoretical and practical reasons^1,2^. On the one hand, they are becoming increasingly more important as materials for the study of transport and charge storage phenomena^3–5^. And on the other hand, there are being successfully and increasingly applied in a wide range of devices, such as electret transducer^6,7^, electret generator^8,9^, energy harvester^10–12^, large-scale flexible electrostatic actuator^13–15^, radiation dosimeter^16^, and so on. Among these, energy harvester is the highlighted focus and has recently gained extensive research interest due to the increasing need of alternative power sources for ubiquitous sensor networks and low power portable devices^17–20^. However, the electrostatic field of electrets may decay in harsh environment, which leads to permanent deterioration of output power performance of energy harvester^21^. Therefore, it has become the top priority to develop electret material with persistent and strong electrostatic effect so as to realize the application of electret-based sensor and actuator with long service life^22^.

Electrets are made from insulating materials. Since the first electret was prepared from carnauba wax and resin with the addition of beeswax in 1919 by Mototaro Eguchi^23^, the development of electret has experienced several important stages. In 1937, Nadjakov obtained the first sulfur electret named as photoelectrets to be distinct from Eguchi’s thermoelectrets^24^. In photoelectrets, the charge can persist for a couple of months in darkness, but vanish rapidly after exposure without applying an electric field. Later, Sessler and West applied thin flexible polymer electrets to microphone in 1962^25^. From then on, perfluoropolymer such as polytetrafluoroethylene (PTFE) and fluorethylenepropylene (FEP) have become the research focus of electrets. PTFE and FEP membranes can be manufactured in different thickness, which makes it possible to be used in various types of transducer devices. However, the charge stored in perfluoropolymer membrane electret would be dispelled when its surface is wiped with alcohol and water in spite that they have excellent charge storage properties in suitable packaging. In order to be compatible with the standard lithogr saphic process of semiconductor, inorganic material represented by SiO_2_ has been chosen as electret in 1983^26^. However, until now, inorganic electret SiO_2_ still cannot implement device application because the space charge stored in SiO_2_ electret is easy to leak out in humid circumstance due to its quick increase of conductance under atmospheric environment^27^. Recently, some new electrets were reported for the improvement of electret performance. For example, Sakane *et al*. proposed a perfluorinated polymer CYTOP electret^28^, Suzuki *et al*. added nano-particles to CYTOP to enhance the trapped charge density^29^; Jun Zhou group proposed polyethylene/carbon nanotubes/T-paper^15^. However, the lasting stability of electret charge storage still remains unrealized completely, and new charge storage mechanism is expected to be put forward. Developing new electret material configuration and preparing the electret material with both persistent and strong electrostatic field is still challenging.

In this paper, a sandwich configuration PTFE/THV/PTFE electret membrane with persistent and strong electrostatic effect is reported. It consists of three layers of perfluoropolymer. The excellent performance of this sandwich electret membrane originates from the synergy of three factors, which are space charge injection, dipole orientation polarization and interfacial polarization. The effect of charging condition on electret performance is explored with surface potential measurement. The capability of resistance to the simulated harsh environment of alcohol wiping is also investigated. Under atmospheric environment and at elevated temperature the stability of electrostatic effect is observed. To assess the electret performance of as-prepared membrane and its resistance to fatigue, a micro-vibration energy harvester is assembled and its performance is detected through a long span of examining test.

## Results and Discussion

### Persistent and strong electrostatic effect of PTFE/THV/PTFE electret membrane

**Figure 1:**
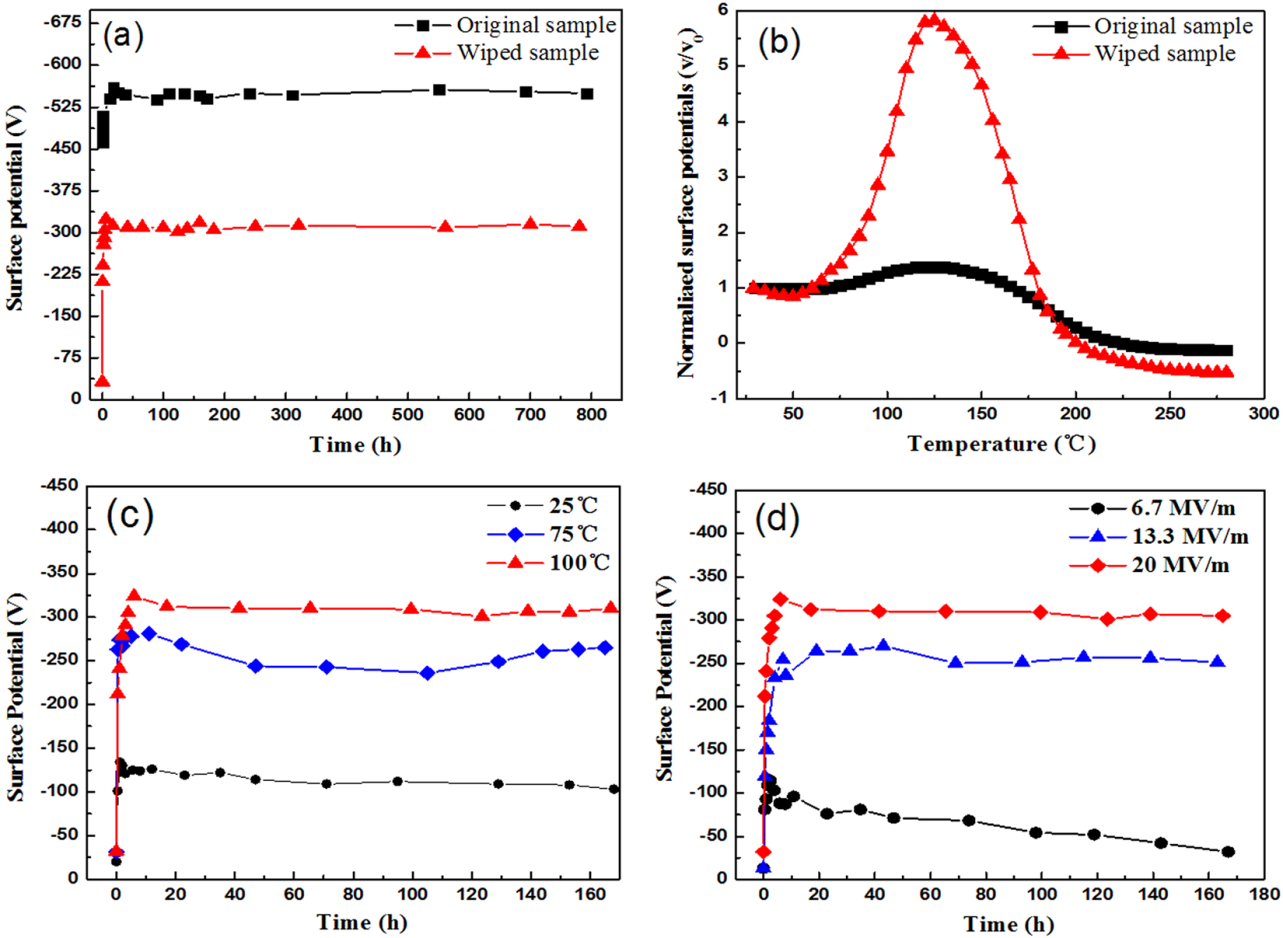
Persistent electrostatic performance of flexible electret membrane. (**a**) *V*_*s*_ of the original and wiped sample as a function of storage time. (**b**) Normalized *V*_*s*_ of the original and wiped sample as a function of temperature. (**c**) *V*_*s*_ as a function of storage time for the wiped sample charged at different *T*_*p*_. (**d**) *V*_*s*_ as a function of storage time for the wiped sample charged at different *E*_*P*_.

**Figure 2:**
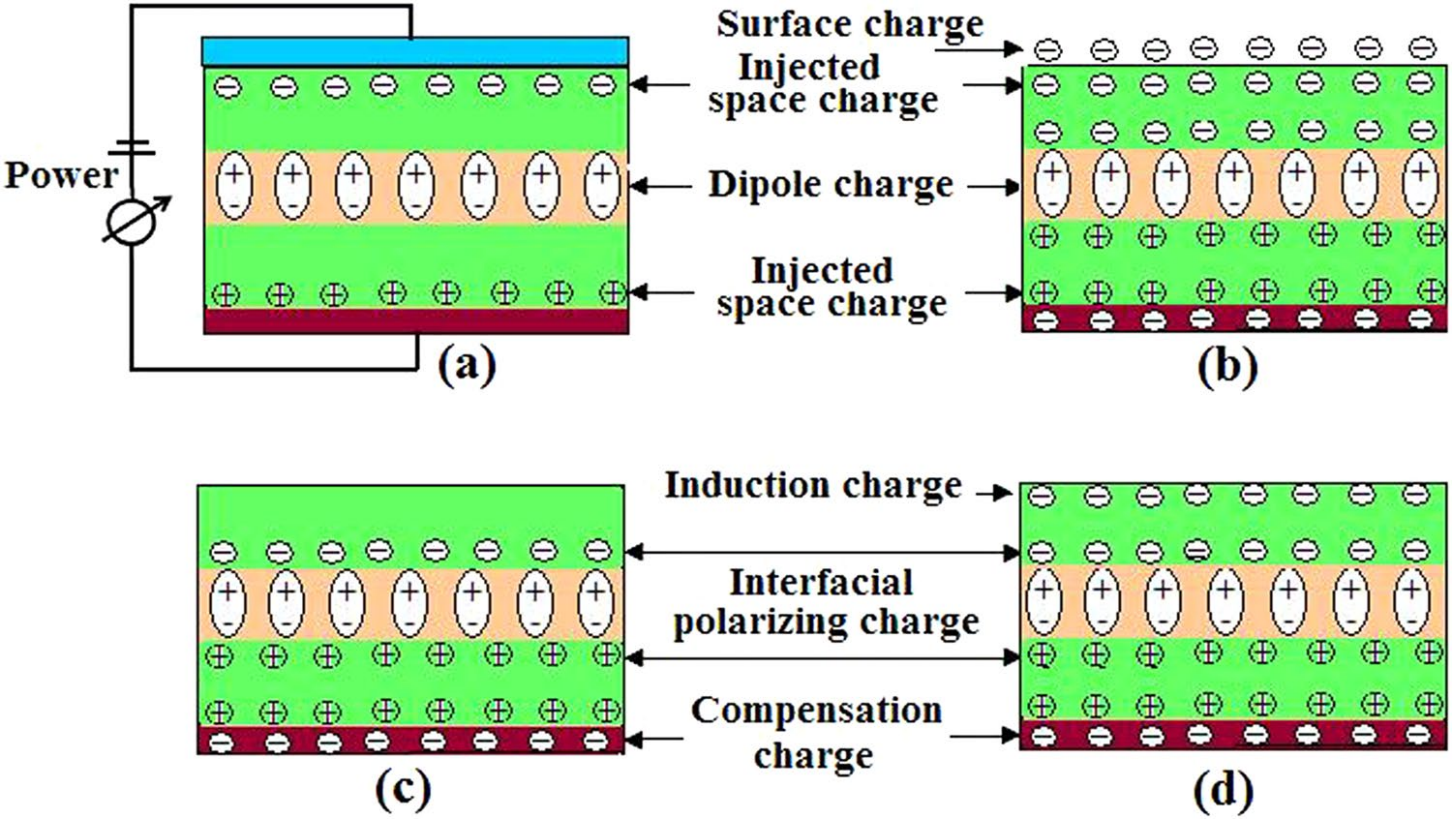
Charge producing and stabilizing mechanism analysis of PTFE/THV/PTFE electret membrane. (**a**) Configuration and charge profile when membrane is charged. (**b**) Charge profile of original sample after electric field is removed. (**c**) Charge profile after surface is cleared with alcohol. (**d**) Charge profile of wiped sample after induction charge is produced.

The electrostatic performance of PTFE/THV/PTFE electret membrane by thermal charging method is displayed in Fig. 1. Figure 1(a) shows the surface potential as a function of storage time after thermal charging at *T*_*p*_ 100 °C and *E*_*P*_ 75 MV m^−1^. This sample is named as the original sample. Its surface potential *V*_*s*_ changes rapidly from −450 V to −520 V within the first 4 hours after charging, and then keeps stable at around −550 V during subsequent storage for 800 hours. In order to notarize the stability of electrostatic effect, a test for charge clearing is implemented. At first, the original sample is charged then stored for 24 hours so as for its *V*_*s*_ to reach stable value of about −550 V. Secondly, sample surface is wiped with pure alcohol to clear off the surface charge so that *V*_*s*_ drops down to zero. And finally, the sample is stored in a dry cabinet under atmospheric environment of 25 °C and 45% relative humidity. This sample is abbreviated as the wiped sample. The surface potential as a function of storage time for the wiped sample is also displayed in Fig. 1(a). It exhibits that *V*_*s*_ recovers rapidly to −310 V within the initial 6 hours after wiping and then remain constant for 800 hours. The results in Fig. 2(a) demonstrates that the original and wiped sample display the same high electrostatic stability while the final stabilized *V*_*s*_ is different. It indicates that the as-prepared PTFE/THV/PTFE electret membrane exhibits terrific resistance to simulated harsh environment such as alcohol wiping. When corona charging method is adopted for sandwich membrane PTFE/THV/PTFE, its initial surface voltage reaches as high as -2325V as shown in Fig. 5(a)﻿. The stable surface voltage is about -1000V after 400-hour storage. However, when wiping the sample surface with alcohol, its surface voltage will recover to about -300V, which is exactly the same as the thermal charged sample. This result indicates that different charging method will lead to the difference of the initial surface charge density for the PTFE/THV/PTFE film, but the most stable charge for PTFE/THV/PTFE is not affected by charging method.

### Distinctive temperature stability of electrostatic effect

The PTFE/THV/PTFE electret membrane also displays distinctive te﻿mperature stability of electrostatic effect under thermal stimulating condition. The original sample is charged at *T*_*p*_ 100 °C and *E*_*P*_ 75 MV m^−1^, and get the initial potential at −583 V before washed with alcohol to wipe off the surface charge. After stored for 24 hours in a dry cabinet, the sample’*s V*_*s*_ recovered to −323 V. A follow-up test of thermal stimulating potential decay is conducted. The thermal stimulating surface potential spectrum, which is normalized as a function of temperature, is plotted in Fig. 1(b). As the temperature rises from room temperature (25 °C) to 280 °C *V*_*s*_ increases rapidly at first and reaches maximum at about 125 °C, where *V*_*s*_ is about 5 times the initial value, and then starts to decline. 125 °C is the peak temperature of thermal stimulating surface potential spectrum. As a comparison, Fig. 1b also shows the thermal stimulating test results for original sample. It shows similar behavior as that of the wiped sample, but the maximum *V*_*s*_ for the wiped sample is dramatically much larger. The PTFE/THV/PTFE sandwich membrane displays excellent temperature stability at 100 °C﻿ as shown in Fig. 5(c), in spite of different charge profile at the initial stage for the original and wiped sample.These results prove that the wiped PTFE/THV/PTFE electret membrane has very good temperature stability.

It is well known that for single-layer PTFE electret membrane, its *V*_*s*_ only displays a decrease profile during thermal stimulating, exhibiting a different trend as PTFE/THV/PTFE electret membrane does. Therefore, this phenomenon of increasing *V*_*s*_ during thermal stimulating test is peculiar for PTFE/THV/PTFE electret membrane. The peak temperature 125 °C corresponding to maximum V_*s*_ in both the original and wiped sample is exactly the softening temperature of the middle layer THV. ﻿Therefore, it can be argued that the surface potential of PTFE/THV/PTFE electret membrane during thermal stimulating is associated with the charge performance of THV layer, in which the decay of dipole orientation polarization occurs as the temperature rises. Fi﻿g. 5(d) shows the comparative results of surface voltage when THV and PVDF are ﻿used as the middle layers of sandwiched structure membrane. THV and PVDF both contain dipoles. The profile of surface voltage exhibits the same variation trend aft﻿er thermal charging. In the initial stage the surface voltage displays a fast increase, and then gra﻿dually maintain constant. This result supports the conclusion that the specific electret performance of sandwiched structure is related to the orientation of internal dipole (dipole charge) within middle layers.

### Effect of charging condition

THV polymer contains TFE, HFP and VDF group, which is the monomer of PTFE, FEP and Poly (vinylidene fluoride) (PVDF), respectively. The VDF polar group is exactly the same as that in the monomer of PVDF. The different ratio of monomer TFE, HFP and VDF will result in different chemical and physical property of THV polymer^30^. In this paper THV has the monomer ratio 45.8:18.5:35.7 (TFE:HFP:VDF) by weight percentage and the softening temperature is about 125 °C. Therefore, orientation and disorientation of VDF group are much easier to occur around its softening temperature 125 °C.

The influence of charging condition on surface potential *V*_*s*_ for wiped sample is investigated. The results are shown in Fig. 1(c) and (d). It displays that *V*_*s*_ increases with the rise of *E*_*P*_ and *T*_*p*_. Under different charging condition, *V*_*s*_ exhibits the similar behavior, which goes through an initial fast increase and then remains constant after about 10 hours. The final stabilized *V*_*s*_ rises with *T*_*p*_ and *E*_*p*_ increasing.

Figure 1(c) shows the influence of charging temperature at *E*_*p*_ 20 MV m^−1^. *V*_*s*_ is only about −125 V when charging at 25 °C. When *T*_*p*_ rise to 75 °C and 100 °C, *V*_*s*_ increases to over −250 V and −300 V, respectively. Figure 1(d) shows the influence of charging electric field at *T*_*p*_ 100 °C. When *E*_*p*_ is 6.7 MV m^−1^, *V*_*s*_ is only about −75 V. As *E*_*p*_ rises to 13.3 and 20.0 MV m^−1^, *V*_*s*_ increases to more than −250 V and −300 V, respectively. These above experimental results further imply that the electrostatic effect of PTFE/THV/PTFE electret membrane is directly associated with the behavior of THV layer, as higher temperature and stronger electric field facilitate the orientation polarization of VDF polar group in THV.

### Analysis of mechanism on charge generating and stabilizing

The electret electrostatic field is caused by the charge stored in the body and on the surface of electret membrane, which is the synergistic effect of three elements, which are external injection, interfacial polarization and orientation of internal dipole (dipole charge). In the light of this idea, the charge profile of PTFE/THV/PTFE electret membrane is theoretically proposed in Fig. 2. Figure 2(a) shows the charge profile while membrane is thermally charged. Space charge is injected to the top and bottom surface of membrane through electrode by Schottky and trap effect. In the meanwhile, the orientation polarization of polar groups in THV layer takes place with the application of charging electric field and dipole charge produces. Figure 2(b) shows the charge profile in the initial 6 hours after the charging electric field is removed. There are three processes to produce charges. One is the interfacial polarization between PTFE layer and THV layer. The other is the appearance of surplus surface charges on top surface of electret membrane after the electric field is removed. And the third is the generation of compensation charge in the bottom electrode. The first two processes lead to the swift increase of *V*_*s*_ during the initial 6 hours after charging, as displayed in Fig. 1. And the last process reduces *V*_*s*_ to almost zero on the bottom surface of electret membrane.

When the top surface of sandwich﻿ed electret membrane is wiped with alcohol, the surface surplus charge and injected shallow trap charge may be wiped off, so that *V*_*s*_ may instantaneously drops to zero. However, they rapidly recover to higher level, as shown in Fig. 1(a,c and d), which is due to the induction of interfacial polarizing charge and dipole charge in middle THV layer.  But for the single layer PTFE, PVDF and THV, the phenomenon of *Vs* recovering don't take place as shown in Fig. 5(b) due to the lack of multiple charge synergies﻿. It should be noted that wiped sample’s *V*_*s*_ couldn’t recover to the same level as that of the original sample. The difference of *V*_*s*_ between the original and wiped sample is about −250 V. This can be attributed to different charge sources according to Fig. 2(c) and 2﻿(d). For the original sample, its *V*_*s*_ originates from the synergistic effect of surface shallow trap charge, surface surplus charge, interfacial polarizing charge and dipole charge. But for the wiped sample, *V*_*s*_ only comes from interfacial polarizing charge and dipole charge, while the surface charge is eliminated in the course of wiping.

As pointed out in the analysis of thermal stimulating discharging, *V*_*s*_ increases when temperature rises and reaches maximum at 125 °C and the wiped sample shows a much larger magnitude than the original sample. It is evidently associated with the dipole disorientation in THV. From Fig. 2(b) and 2 (d), as temperature rises, the dipole disorientation gradually appears and reaches its peak at the softening temperature 125 °C of THV, which leads to the decay of positive charge in the body of electret membrane and the addition of the negative charge on the bottom surface of electret membrane.

### Performance of micro-vibration energy harvester assembled with PTFE/THV/PTFE electret membrane

**Figure 3:**
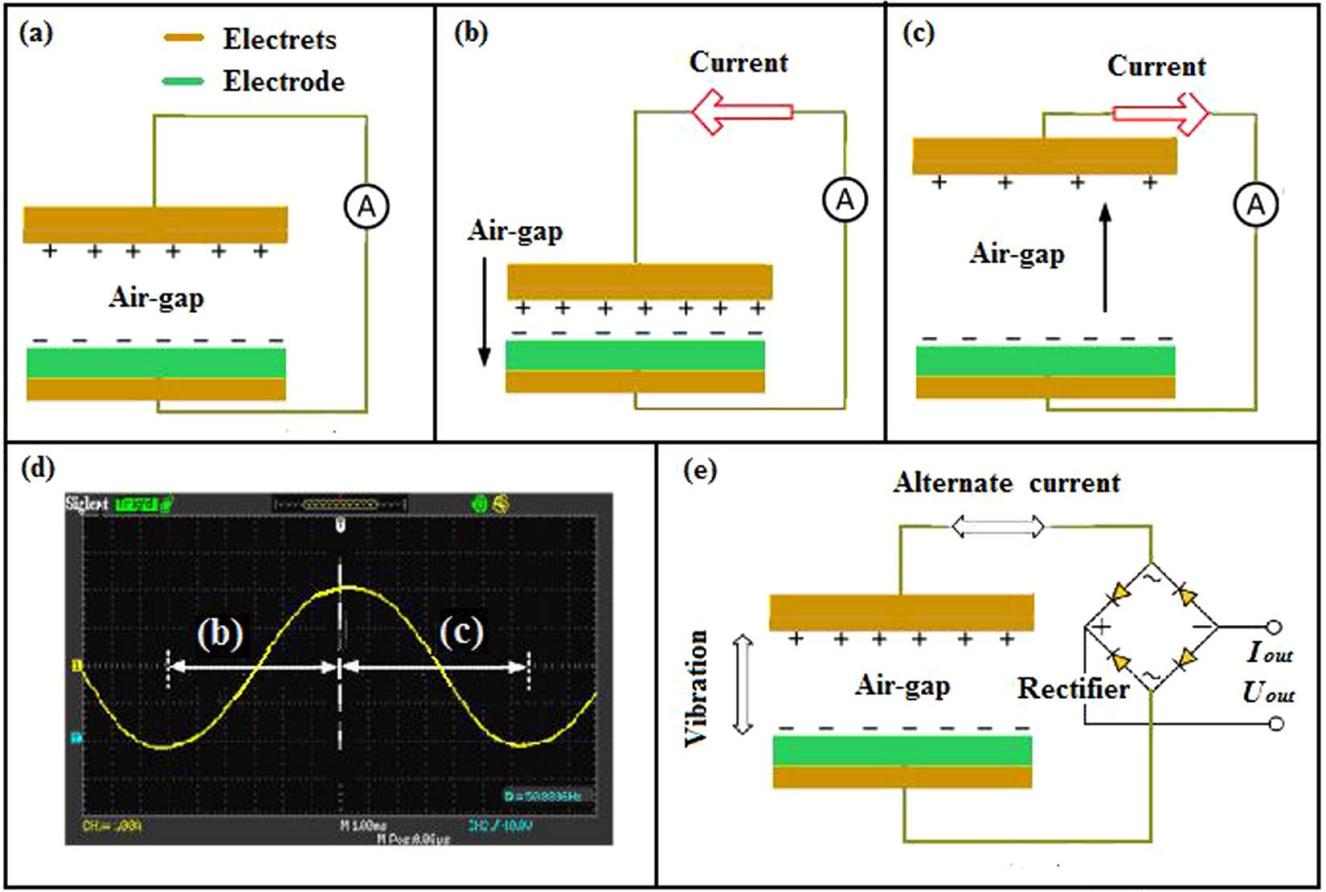
Configuration schematic and current output performance of vertical vibration energy harvester. (**a**) Basic configuration of energy harvester; (**b**) Current output when air-gap is reduced; (**c**) Current output when air-gap is added; (**d**) Waveform of current output corresponding to (**b**) and (**c**) process; (**e**) Entire configuration of vertical vibration energy harvester.

To investigate the persistent and strong electrostatic effect of PTFE/THV/PTFE electret membrane, a micro-vibration energy harvester is prepared with this electret membrane. Its configuration schematic and current output performance is shown in Fig. 3. The energy harvester consists of an electret membrane metalized on one surface (also used as bottom electrode) and a top electrode^31–33^. The bottom electrode is fixed by a holding-fixture, and the top electrode is fixed on a vibrator vertically vibrating in a controllable manner. Negatively charged surface of electret membrane is faced to the top electrode. The capacitance varies with the air-gap distance between the top electrode and the electret membrane, which generates an alternating current. This alternating current can be changed into direct current through a full bridge rectifier, and is recorded with an electrometer (Keithley 6517B, USA).

Initial air-gap distance between top electrode and electret membrane is set to 5 mm. The steady *V*_*s*_ of electret membrane is −588 V, which gives the surface charge density σ of 1.43 μC m^−2^ calculated from formula *ε*_*r*_*ε*_*0*_*V*_*s*_*/g. ε*_r_ is the relative permittivity of electret material, *ε*_*0*_ is the vacuum permittivity, *g* is the thickness of electret membrane, respectively. The area of top electrode and electret membrane is both 3 cm × 3 cm. When top electrode is continuously tapped, output voltage and current can be read on an electrometer. The output characteristics of this energy harvester are shown in Table 1. It exhibits that output voltage and current both rise with the increase of tapping frequency, therefore the output power also increases. The output power reaches 4.66 μW when tapping frequency is 5 Hz. This experimental result demonstrates that the PTFE/THV/PTFE electret membrane has very strong electrostatic effect.

**Table 1:** Output characteristics of energy harvester assembled with PTFE/THV/PTFE electret membrane.

Tapping frequency [Hz]	Output voltage [V]	Output current [μA]	Output power [μW]
1	11.6	0.021	0.24
3	17.7	0.041	0.73
5	23.3	0.12	4.66

It is worth noted that the output characteristics of this energy harvester keeps constant through over 2000 tapping tests and no decay occurs during 100-day test process. This result further proves that PTFE/THV/PTFE electret membrane has superb electrostatic effect stability and distinctive capability of resistance to fatigue. In accordance with these above experimental results, long-life service of energy harvester assembled by this electret membrane can be predicted affirmatively.

## Conclusion

A flexible electret membrane consisting of three-layer perfluoropolymer exhibiting persistent and strong electrostatic effect, in which the top and bottom layers are PTFE and the middle layer is THV, can be prepared by means of hot pressing method and thermal charging technology. A constant electrostatic field of about −550 V lasting for 800 hours is observed under the atmospheric condition. Experimental result of wiping electret membrane surface with alcohol demonstrates that a stable electrostatic field of about −310 V can be recovered after surface charge being wiped off completely and thereafter this stable field can be maintained for 800 hours. The measurement of thermal stimulating potential decay at elevated temperature demonstrates that PTFE/THV/PTFE electret membrane has outstanding temperature stability. Its *V*_*s*_ increases rapidly at first and reaches maximum about 5 times the initial value at about 125 °C. The stability of electrostatic effect is correlated to the softening temperature of THV polymer layer. Moreover, the study of charging condition further implies that the stable electrostatic effect is directly associated with the behavior of THV layer.

Based on three factors of electret charge producing, which are space charge injection, dipole orientation and interfacial polarization, a charge profile is proposed. The persistent electrostatic effect of PTFE/THV/PTFE electret membrane is correlated to the synergy of these above three factors. What is worth mentioning, the behavior of VDF polar group in THV layer plays a very important role in this synergy effect.

A micro-vibration energy harvester is assembled with the PTFE/THV/PTFE electret membrane. Its output power reaches 4.66 μW with the tapping frequency of 5 Hz. The output characteristics of this energy harvester keep constant during over 2000 tapping tests within the 100-day test process. This result further proves that the PTFE/THV/PTFE electret membrane has excellent electrostatic effect stability and distinctive capability of resistance to harsh environment and fatigue as well. Therefore, it indicates that the energy harvester with long-life service can be obtained in the near future.

## Experimental procedure

As shown in Fig. 1, this flexible electret membrane consists of three layers of perfluoropolymer and is abbreviated as PTFE/THV/PTFE, where the top and the bottom layers are polytetrafluoroethylene (PTFE) membrane, and the middle layer is fluoride terpolymer THV [tetrafluoroethylene (TFE)-Hexafluoropropylene (HFP) -Vinylidene (VDF)]. It is prepared as follows. Firstly, three layers of perfluoropolymer are heated for 10 minutes at elevated temperature and under high pressure so as to be adhered to each other; Secondly, the adhesive sandwich membrane is metalized with aluminum on the bottom surface and cut into 3 cm × 3 cm squares. All membranes are wiped with alcohol before test. A 50 µm thick PTFE membrane is commercially available from Du Pont Co. The THV membrane of 50 µm thickness is thermoformed from THV powder of Dyneon Co.

After the aluminum electrode is evaporated on the bottom surface of PTFE/THV/PTFE membrane in vacuum, the metalized sandwich membrane is charged by means of thermal charging method as illustrated in Fig. 1(d) under atmospheric pressure, relative humidity of 45% and at elevated temperature. The aluminized surface is placed on an electrically grounded metal plate with an electric field applied at elevated temperature. After charged, the electret samples are naturally cooled down to room temperature (25 °C) while maintaining applied electric field *E*_*P*_, then moved out from charging set-up and stored in a dry cabinet at room temperature and relative humidity of 45%. The charging condition is as follows unless specially pointed out: electric field (*E*_*P*_) 75 V m^−1^, charging time 10 minutes and charging temperature (*T*_*p*_) 100 °C.

**Figure 4:**
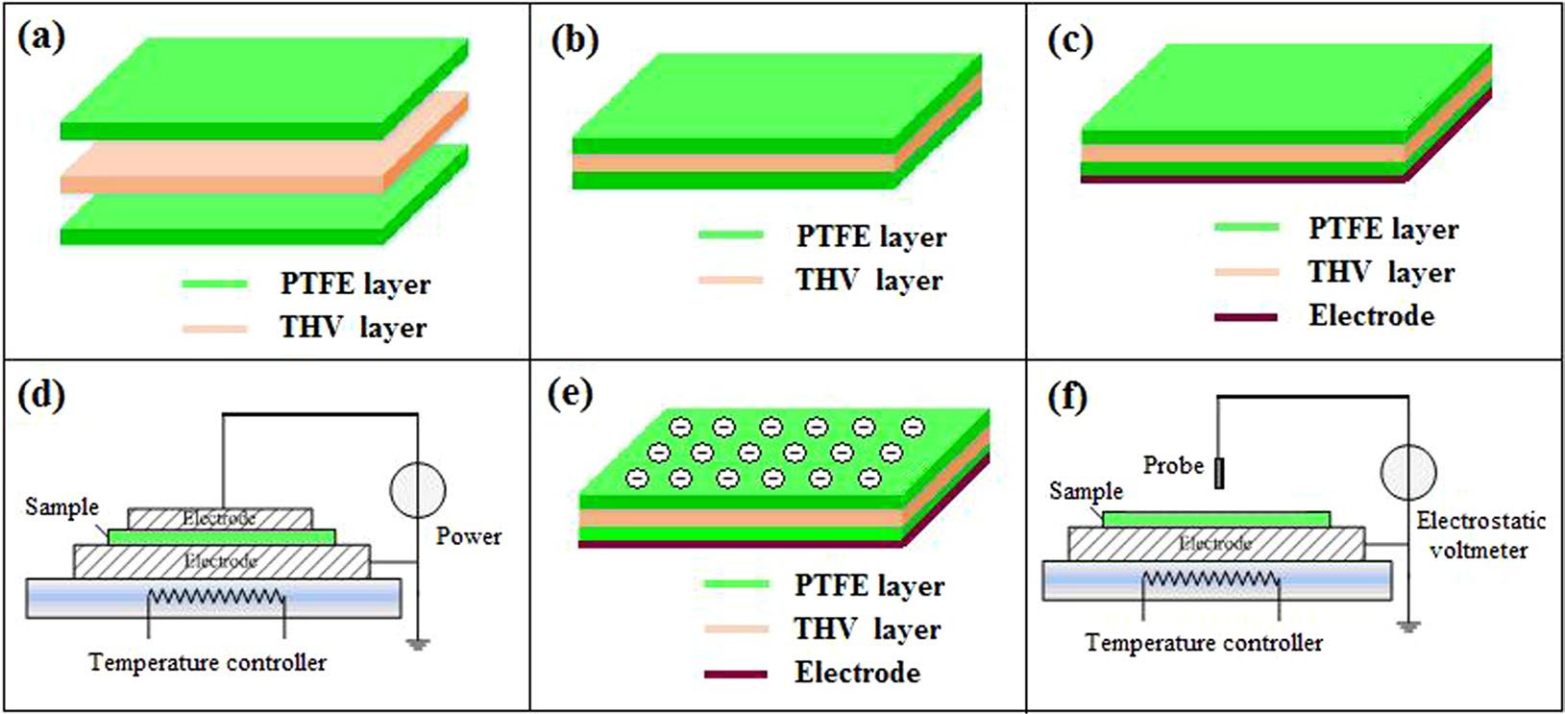
Illustration of flexible PTFE/THV/PTFE electret membrane preparation. (**a**) Constitution of PTFE/THV/PTFE membrane; (**b**) Structure of hot pressed membrane; (**c**) Metalized membrane on the bottom surface with aluminum; (**d**) Configuration schematic of thermal charging; (**e**) Surface charge distribution of electret membrane; (**f**) Configuration schematic of surface potential measurement.

As illustrated in Fig. 4(﻿f), the stability of electrostatic field for PTFE/THV/PTFE electret membrane is determined by measuring surface potential *V*_*s*_ with non-contact field-compensating electrostatic voltmeters (Monroe 244, USA). For the measurement of thermal stimulating surface potential decay, the sample is put upon a heater with temperature controller. Heating rate was 6 °C/min from room temperature to 140 °C and *V*_*s*_ is recorded at an interval of 10 °C.

**Figure 5:**
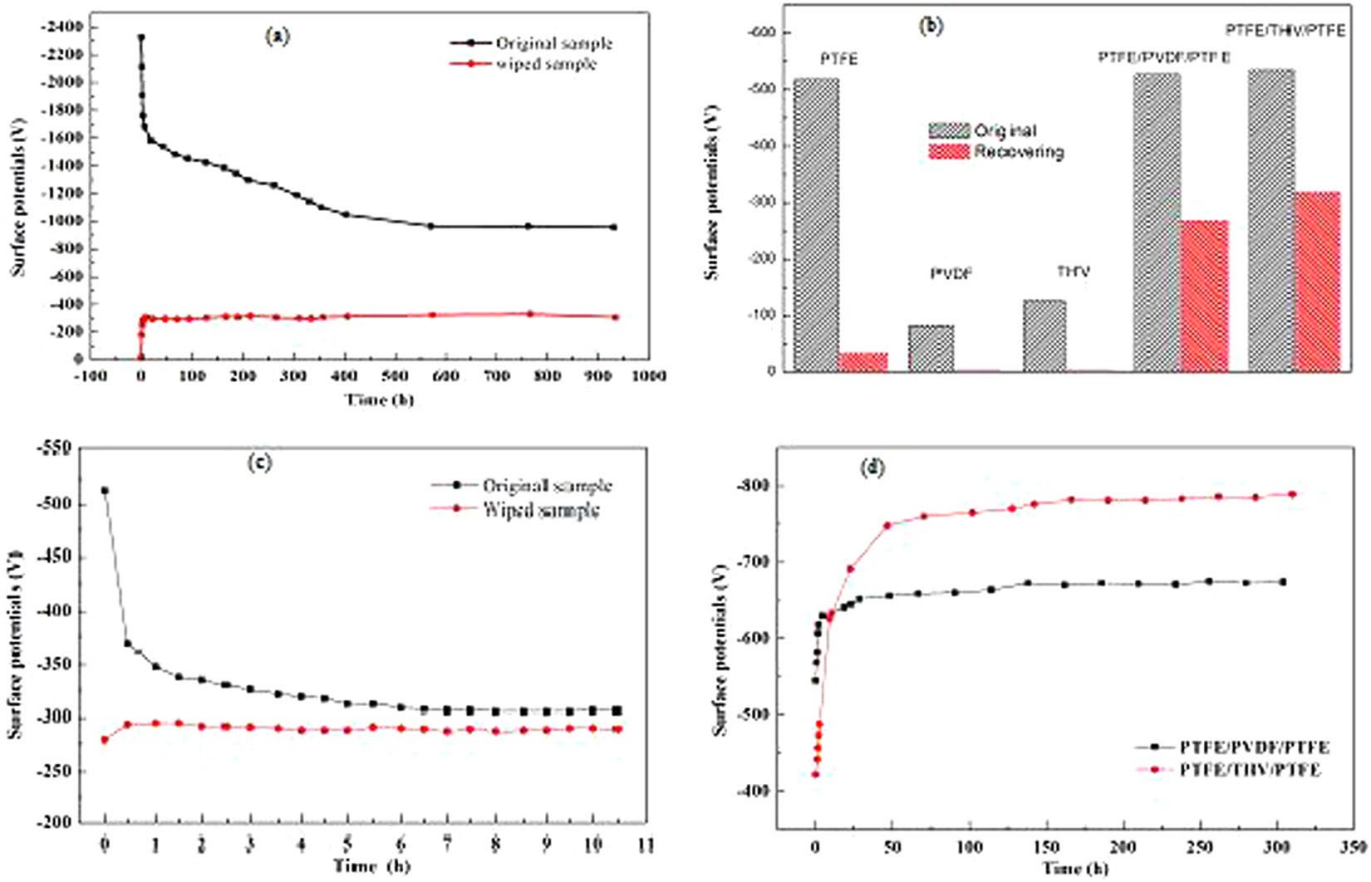
Supplementary experimental results (different﻿ charging me﻿thods,﻿ comparison with single layers and sandwiched layers, different middle layers). ﻿**(a)***V*_S_ ﻿of the origi﻿nal and wiped PTFE/THV/PTFE as a function of storage time by corona charging method; **(b)***V*_S_ self-recovery performance for single layer (PTFE, PVDF, THV) and sandwich membrane (PTFE/PVDF/PTFE, PTFE/THV/PTFE). Grey column: original state; Red column: recovering state after wiping sample surface with alcohol and storing in atmosphere for 24 hours; **(c)***V*_S_ ﻿of the original and wiped PTFE/THV/PTFE as a function of storage time at storage temperature 100 °C by thermal charging method; **(d)** *V*_S_ of the sandwich membrane ﻿with different middle layers PVDF and﻿ THV as a function of storage time.

## Electronic supplementary material

Supplementary document for video

Energy harvesting process
