# Author Correction: A Flexible Electret Membrane with Persistent Electrostatic Effect and Resistance to Harsh Environment for Energy Harvesting

**DOI:** 10.1038/s41598-018-23230-8

**Published:** 2018-03-22

**Authors:** Xiao Huiming, Chen Gangjin, Chen Xumin, Chen Zhi

**Affiliations:** 0000 0000 9804 6672 grid.411963.8 Lab. of Electret and Its Application, Hangzhou Dianzi University, Hangzhou, 310018 China

Correction to: *Scientific Reports* 10.1038/s41598-017-07747-y, published online 16 August 2017

The original version of this Article contained a typographical error in the spelling of the author Chen Xumin, which was incorrectly given as Chen Xuming. This error has now been corrected in the PDF and HTML versions of the Article, and in the accompanying Supplementary document.

