# Supplementary material for: A Flexible Electret Membrane with Persistent Electrostatic Effect and Resistance to Harsh Environment for Energy Harvesting
Source: Sci Rep. 2017 Aug 16;7:8443. doi: 10.1038/s41598-017-07747-y (PMC5559523; doi:10.1038/s41598-017-07747-y)
Supplement: Supplementary file 1 — Supplementary document for video [file 41598_2017_7747_MOESM1_ESM.docx]

A Flexible Electret Membrane with Persistent Electrostatic Effect and Resistance to Harsh Environment for Energy Harvesting

**Xiao Huiming, Chen Gangjin,** **Chen Xumin, Chen** **Zhi**

Lab. of Electret and Its Application, Hangzhou Dianzi University, Hangzhou, China, 310018

Correspondence and requests for materials should be addressed to C.G. (email: cgjin@hdu.edu.cn)
